# Pupil size changes reveal dogs’ sensitivity to motion cues

**DOI:** 10.1016/j.isci.2022.104801

**Published:** 2022-07-20

**Authors:** Christoph J. Völter, Ludwig Huber

**Affiliations:** 1 Comparative Cognition, Messerli Research Institute, University of Veterinary Medicine Vienna, Medical University of Vienna and University of Vienna, Veterinaerplatz 1, 1210 Vienna, Austria

**Keywords:** Animals, Ethology, Cognitive neuroscience, Animal of veterinary interest

## Abstract

Certain motion cues like self-propulsion and speed changes allow human and nonhuman animals to quickly detect animate beings. In the current eye-tracking study, we examined whether dogs’ (*Canis familiaris*) pupil size was influenced by such motion cues. In Experiment 1, dogs watched different videos with normal or reversed playback direction showing a human agent releasing an object. The reversed playback gave the impression that the objects were self-propelled. In Experiment 2, dogs watched videos of a rolling ball that either moved at constant or variable speed. We found that the dogs’ pupil size only changed significantly over the course of the videos in the conditions with self-propelled (upward) movements (Experiment 1) or variable speed (Experiment 2). Our findings suggest that dogs orient toward self-propelled stimuli that move at variable speed, which might contribute to their detection of animate beings.

## Highlights

• We applied eye tracking and pupillometry to study dogs’ animacy perception
• Dogs oriented toward self-propelled stimuli that moved at variable speed
• Dogs’ pupil size was more variable when presented with these motion cues
• Orienting to such motion cues might support dogs’ detection of animate beings

## Graphical abstract

**Figure:**
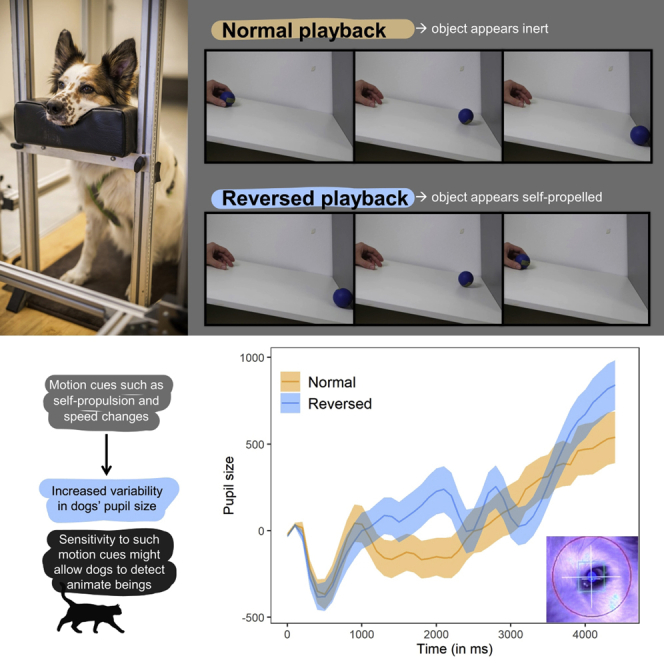

## Introduction

Certain motion cues allow humans and nonhuman animals to efficiently detect animate beings (e.g., Rosa-Salva et al., 2016; Scholl and Tremoulet, 2000; Tremoulet and Feldman, 2000). These so-called animacy cues include self-propulsion (i.e. movement without any obvious external cause), nonlinear movement patterns, and direction or speed changes that are characteristic of animate beings. There is evidence that infants from an early age are sensitive to these cues (e.g., Csibra et al., 1999; Di Giorgio et al., 2021; Frankenhuis et al., 2013; Gergely et al., 1995; Premack, 1990; Rochat et al., 1997; Schlottmann and Surian, 1999; Scholl and Tremoulet, 2000; Träuble et al., 2014).

The “energy violation” hypothesis of animacy perception in humans suggests that moving bodies are perceived as animate when their motion would require a hidden energy resource (Dittrich and Lea, 1994; Scholl and Tremoulet, 2000). This hypothesis predicts that motion cues such as speed and direction changes and movements that defy gravity in the absence of external causes might lead to a perception of animacy. Indeed, there is evidence that humans judge upward moving dots more often as animate than downward moving dots (Szego and Rutherford, 2008).

Surprisingly, only few studies looked into animacy perception in nonhuman animals: Three-month-old Japanese macaques (*Macaca fuscata*) showed a looking time preference for a self-propelled object (a stone) over a still one (Tsutsumi et al., 2012). Adding artificial fur to the stone increased the looking times also in the still condition, and no difference between the still and self-propelled conditions was found any more. Adult cotton-top tamarins’ (*Saguinus oedipus oedipus*) looking times were longer when inanimate objects changed their location while being occluded behind a screen but not when live animals (a mouse or a frog) or a self-propelled, furry toy mouse changed location (Hauser, 1998). These findings suggest that both the combination of fur and self-propelled motion might contribute to animacy perception in primates. Outside the primate order, there is evidence that newborn chicks distinguish between self-propelled objects (moving without apparent external cause) and moving objects that were launched by another object (Mascalzoni et al., 2010). The chicks preferentially approached and imprinted on the self-propelled object. Another study found that chicks also preferred self-propelled objects that moved at a changing speed over an object moving at a constant speed (Rosa-Salva et al., 2016). Other studies used a predator detection context to study animacy perception: Breeding jackdaws (*Corvus monedula*) reacted with a stronger startle response to self-propelled objects than still ones although the effect was modulated by the object appearance (sticks versus toy animals) (Greggor et al., 2018). Finally, there is some evidence with fish: fathead minnows (*Pimephales promelas*) reacted aversively to self-propelled objects but not to still objects with a fish-like shape (Wisenden and Harter, 2001). Together these studies provide some, albeit patchy, evidence that some animal species are sensitive to animacy cues such as self-propulsion and speed changes that might help them to detect conspecifics and predators.

Locating and identifying prey is another function of animacy perception. Studying carnivores, in particular social carnivores such as dogs (*Canis familiaris*) that also need to detect social partners, is therefore of particular interest in this context. Indeed, a number of studies investigated animacy perception in dogs, mainly in three different ways: by presenting them with (1) biological motion cues (point-light animations), (2) dependent (i.e. chasing-like) and independent movement patterns, and (3) nonlinear, changing movement trajectories. First, biological motion cues are usually studied using moving dots that show the movement pattern of a walking human or of another animal. In one study, dogs were found to look longer at a human point-light walker (shown in lateral view) than a scrambled-points control (Kovács et al., 2016). This finding, however, could not be replicated: Ishikawa et al. (2018) reported that dogs looked longer at a human point-light walker in frontal, but not lateral, view (in comparison to a control condition with inverted points). They found no preference for dog point-light walkers. Eatherington et al. (2019) found no effect for lateral human point-light walkers but the dogs showed increased looking times to upright dog point-light walkers than inverted ones (however, there was no difference when compared with scrambled stimuli). Dogs also failed to follow a human pointing gesture just based on a point-light walker display (Eatherington et al., 2021). How dogs perceive biological motion cues remains therefore controversial (at least in the context of point-light stimuli).

Secondly, when presented with two animations (shown simultaneously, side-by-side) of two moving stimuli (dots or triangles) that either displayed a dependent (one stimulus following the other one in a chasing-like manner) or independent movement pattern, dogs initially preferentially looked at the chasing motion pattern (Abdai et al., 2020) or showed no difference between the two patterns (Abdai et al., 2017b). With some experience they preferred looking at the independent movement pattern (Abdai et al., 2017b, 2020). When presenting dogs with two real objects (remote-controlled toy cars and not animations of simple geometric shapes), dogs, however, preferred to approach objects that showed a dependent movement pattern (Abdai et al., 2017a). The dogs’ different reactions across the two paradigms might be explained by differences in the stimulus presentation. In the case of projected stimuli (Abdai et al., 2017b, 2020), the dogs’ looking times might have reflected recognition of a chasing event involving two animate beings, whereas their performance with real objects (Abdai et al., 2017a) might have reflected a preference for approaching and interacting with animate beings (e.g., prey).

Third, when presented with self-propelled objects that changed the movement trajectory either before bumping into an obstacle or only after bumping into it, dogs more often approached the object that avoided the collision (Tauzin et al., 2017). Thus, dogs chose to explore objects that changed direction without external cause. In line with this finding, we recently found that dogs show increased pupil sizes in response to an event in which a rolling ball suddenly stopped and another ball started moving without contact between the two (i.e. the balls moved as if one ball was launching the other but without ever making contact) compared with a regular launching event with the same timing and kinematic properties (Völter and Huber, 2021a).

Despite some failed replications (particularly with respect to biological motion perception), there is evidence that dogs’ perception is attuned to certain animacy cues, particularly dependent motion patterns and changes in the movement trajectory. One reason for the heterogeneous findings might be that the looking times and object choice in this context might be quite variable across individuals and potentially susceptible to (seemingly) minor variations in the task presentation. Another reason might be that measuring looking times based on manual video scorings can be imprecise. The current study advances the study of dogs’ animacy perception by applying eye tracking technology, focusing on pupillometry as main response variable, and by looking at additional cues including speed changes, the stimulus appearance (presence of fur), and self-propelled movements that defy gravity.

Changes in pupil sizes are caused by different factors; the most important ones being the pupillary light response to the environmental light conditions and the pupil near response to close fixations (Mathôt, 2018). When these other factors can be controlled, pupillometry is a widely used measure for an orienting response, cognitive load, and arousal in humans, and there are data with primates and other mammals such as mice (Lee and Margolis, 2016) suggesting that this relationship might be common to mammals. As for dogs, there is already some first evidence that pupil size can be used to measure arousal: dogs exhibited larger pupils when presented with human faces with an angry emotional expression (Karl et al., 2020b; Somppi et al., 2017) and we recently found evidence that pupillometry can indicate expectancy violations in dogs (Völter and Huber, 2021a). Although there is no research yet investigating the relation between animacy cues and pupil dilation, there is at least anecdotal evidence from other carnivore species, namely cats, that motion cues in play and hunting contexts can lead to a pupil dilation response (Hess and Polt, 1960). In the current study, our aim was to investigate whether motion cues associated with animacy perception in humans would lead to a pupil dilation response in dogs.

Watching reverse playing videos has a captivating effect on humans, an effect exploited by cinema films such as “Tenet” (Nolan, 2020). One reason might be that the reversed playback direction can induce animacy cues, namely that object movements appear as self-propelled rather than inert or are caused by an external event or force (e.g., a launching or dropping event). In addition, actions or object movements can defy physical laws such as gravity when played back in reverse. According to the energy violation hypothesis, objects that move as if they have access to a hidden energy source can lead to a perception of animacy (Dittrich and Lea, 1994; Scholl and Tremoulet, 2000). In the first experiment, we utilized this effect and examined how dogs would react to videos with normal or reversed playback direction. In a second experiment, we presented dogs with controlled animations of a stimulus (smooth ball or fur ball) moving along a straight, horizontal path. Apart from the appearance of the stimulus, we varied whether the object moved with constant or variable speed. In line with research with humans (Scholl and Tremoulet, 2000; Träuble et al., 2014), new born chicks (Rosa-Salva et al., 2016), and previous research with dogs (Abdai et al., 2017a, 2017b, 2020; Tauzin et al., 2017; Völter and Huber, 2021a), we predicted that the dogs would be sensitive to self-propulsion and speed changes. In addition, we expected that dogs would look longer at the self-propelled objects (videos with reversed playback direction) and objects that move with variable speed at the end of the video when the object did not move any longer.

## Results

### Experiment 1

In Experiment 1, we tracked the dogs’ eye movements and their pupil size while they watched one out of three videos (Cube, Ball, and Rope; see Video S1), depending on the session either with the normal or reversed playback direction.

Video S1. Video stimuli presented in Experiment 1 with the gaze of a dog overlaid, related to STAR Methods

#### Cube video

**Figure 1:**
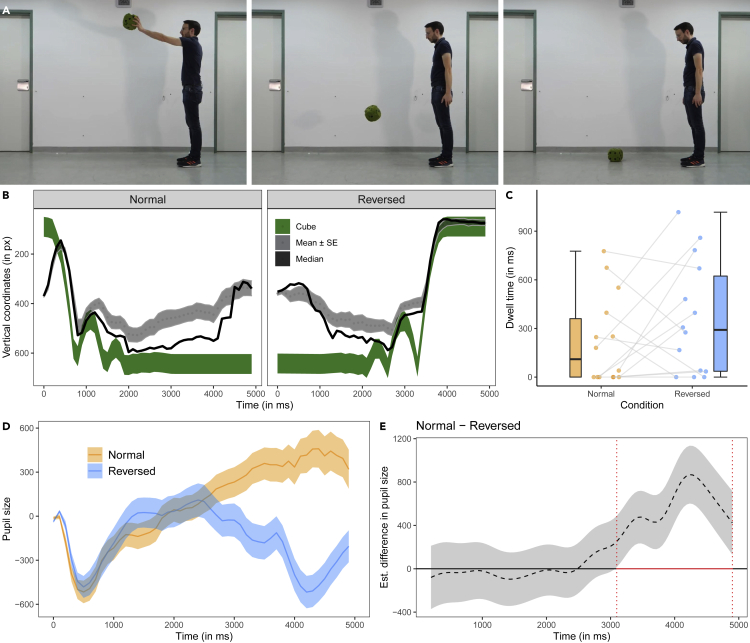
Screenshots and data visualizations of Experiment 1a (A) Screenshots of the cube video (at 0, 1.1, and 4.0 s of the forward playing video) that was either played forward or reverse. (B) Time series plot showing the dogs’ median (black line) and mean vertical gaze coordinates (±se, dotted line and the dark gray shaded area around it; in px) in the test trials. The shaded green area represents the moving cube. (C) Boxplot showing the dogs’ looking times to the areas of interest around the cube’s end position at the end of the video. The dots represent the mean individual values. (D) Time series plot showing dogs' pupil size (in arbitrary units and baseline corrected). The orange and blue lines show the mean pupil size (±se, shaded area around the line) in the Normal and Reversed condition. (E) Difference curve derived from a GAMM. The dashed line shows the estimated difference between the Normal and Reversed condition; the shaded area shows the pointwise 95% confidence interval. The period in which the conditions differ significantly is highlighted in red.

The cube video (see Figure 1A) showed an actor holding a foam cube in his outstretched hand and then dropping it to the ground. When it hit the ground, it bounced off twice before coming to a rest. In both the Normal and Reversed playback conditions, the vertical movements of the cube explained a substantial amount of variance in dogs’ vertical eye movements (mean r^2^ ± se, Normal: r^2^ = 0.32 ± 0.01; Reversed: mean r^2^ = 0.46 ± 0.02; Figure 1B). The interest area (IA) analysis of the interest period at the end of the video (with the cube in its respective end position) revealed no significant difference between conditions (t(13) = −1.36, p = 0.198; Figure 1C). There were no significant differences in the average fixation duration or the fixation count across conditions but the dogs made saccades with (on average) larger amplitudes in the normal condition compared with reversed condition (see Table S1). This finding is in line with the finding that the dogs followed the vertical movements of the cube more accurately in the reversed condition than the normal condition. During the entire trial there was no significant difference between the dwell times to the actor’s head IA (t(13) = 1.49, p = 0.160) or the mean number of IA visits (including lower cube position IA, upper cube position IA, and actor’s head IA; t(13) = 1.40, p = 0.185) that could explain the difference in motion tracking between conditions.

We fitted a generalized additive mixed model (GAMM) to the preprocessed pupil size data. In this model (GAMM 01 and the following ones), we included condition and order of condition as parametric terms, the nonparametric regression lines for time and for the two levels of condition over time, and the nonlinear interaction between X and Y gaze positions (because previous research has shown that the gaze position can affect the pupil size; Mathôt et al., 2018; van Rij et al., 2019). The comparison between GAMM 01 and a respective null model without the parametric and nonparametric effects of condition revealed that condition significantly improved the model fit (Chi-square test of ML scores: χ^2^(5) = 89.66, p < 0.001; GAMM01 had a lower AIC: ΔAIC 143.87). The model summary revealed a significantly larger pupil size in the normal compared with the reversed condition (t = −2.01, p = 0.044) and a significant change of the pupil size over time in the Reversed condition (F(15.49, 16.48) = 5.28, p < 0.001) but not the Normal condition (F(4.85, 5.34) = 0.44, p = 0.841). The difference curve confirmed that dogs had larger pupils in the Normal than Reversed condition in the time window between 3,096 and 4,900 ms (Figures 1D and 1E; for the model estimates, partial and summed effects see Table S2 and Figure S3).

#### Ball video

**Figure 2:**
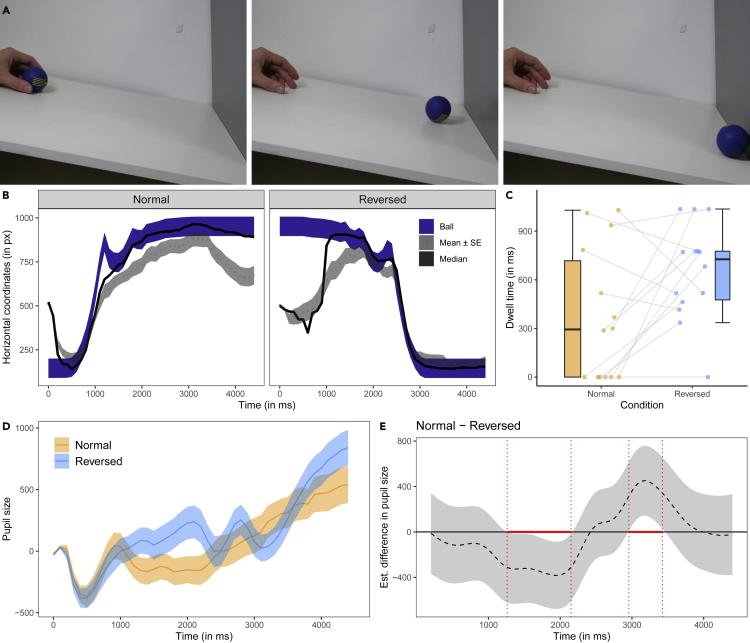
Screenshots and data visualizations of Experiment 1b (A) Screenshots of the ball video (at 0, 1.3, and 3.5 s of the forward playing video) that was either played forward or reverse. (B) Time series plot showing the dogs’ median (black line) and mean horizontal gaze coordinates (±se, dotted line and the dark gray shaded area around it; in px) in the test trials. The shaded blue area represents the moving ball. (C) Boxplot showing the dogs’ looking times to the areas of interest around the ball’s end position at the end of the video. The dots represent the mean individual values. (D) Time series plot showing dogs' pupil size (in arbitrary units and baseline corrected). The orange and blue lines show the mean pupil size (±se, shaded area around the line) in the Normal and Reversed condition. (E) Difference curve derived from a GAMM. The dashed line shows the estimated difference between the Normal and Reversed condition; the shaded area shows the pointwise 95% confidence interval. The period in which the conditions differ significantly is highlighted in red.

In the ball video (see Figure 2A), a human hand was shown to release a ball at the upper end of a slanted surface. The ball rolled down the surface where it hit a vertical wall from which it bounced off twice before coming to a rest. In both Normal and Reversed playback conditions, the horizontal movements of the ball explained a substantial amount of variance in dogs’ horizontal eye movements (mean r^2^ ± se, Normal: r^2^ = 0.65 ± 0.02; Reversed: mean r^2^ = 0.49 ± 0.02; Figure 2B). The IA analysis of the interest period at the end of the video (with the ball in its end position) showed that the dogs looked longer at the ball in the Reversed condition than the Normal condition (t(13) = −2.28, p = 0.040; Figure 2C). There were no significant differences in the average fixation duration, the fixation count, or the mean saccade amplitude across conditions (see Table S3).

The comparison between GAMM 02 and a respective null model without the parametric and nonparametric effects of condition revealed that condition significantly improved the model fit (Chi-square test of ML scores: χ^2^(5) = 70.87, p < 0.001; GAMM02 had a lower AIC: ΔAIC 129.54). The model summary showed a significant change of the pupil size over time in the Reversed condition (F(10.80, 11.81) = 2.03, p = 0.040) but not the Normal condition (F(9.32, 10.13) = 0.38, p = 0.879). The difference curve revealed that dogs had larger pupils in the Reversed than Normal condition in the time window between 1,261 and 2,152 ms. Between 2,958 and 3,424 ms, their pupils were larger in the Normal than Reversed condition (Figures 2D and 2E; for the model estimates and partial and summed effects see Table S4 and Figure S2).

#### Rope video

**Figure 3:**
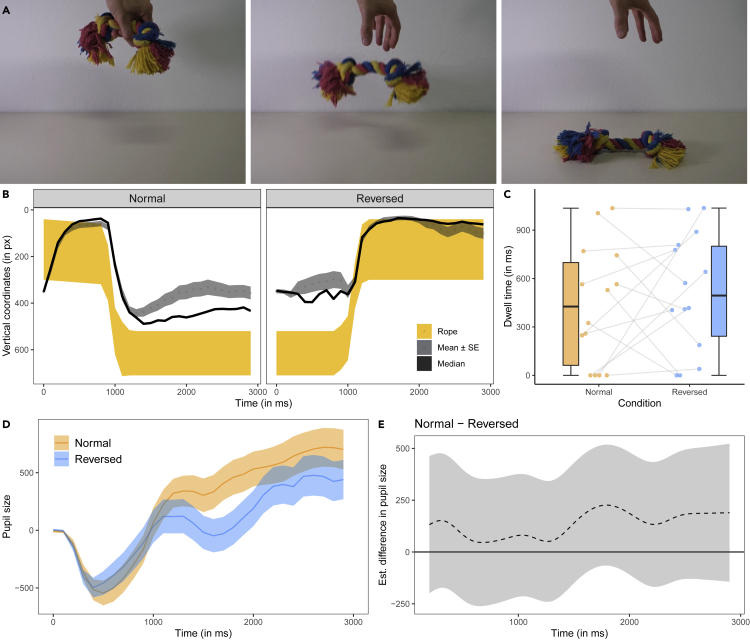
Screenshots and data visualizations of Experiment 1c (A) Screenshots of the rope video (at 0, 0.9, and 2.0 s of the forward playing video) that was either played forward or reverse. (B) Time series plot showing the dogs’ median (black line) and mean horizontal gaze coordinates (±se, dotted line and the dark gray shaded area around it; in px) in the test trials. The shaded yellow area represents the moving rope. (C) Boxplot showing the dogs’ looking times to the areas of interest around the rope’s end position at the end of the video. The dots represent the mean individual values. (D) Time series plot showing dogs' pupil size (in arbitrary units and baseline corrected). The orange and blue lines show the mean pupil size (±se, shaded area around the line) in the Normal and Reversed condition. (E) Difference curve derived from a GAMM. The dashed line shows the estimated difference between the Normal and Reversed condition, the shaded area shows the pointwise 95% confidence interval.

In the rope video (see Figure 3A), a human hand holding a short piece of rope was shown to release the rope that then fell down to the ground. In both conditions, the vertical movements of the cube explained a substantial amount of variance in dogs’ vertical eye movements (mean r^2^ ± se, Normal: r^2^ = 0.59 ± 0.02; Reversed: mean r^2^ = 0.58 ± 0.02; Figure 3B). The IA analysis of the interest period at the end of the video (with the cube in its respective end position) revealed no significant difference between conditions (t(13) = −0.66, p = 0.523; Figure 3C). There were no significant differences in the average fixation duration, the fixation count, or the mean saccade amplitude across conditions (see Table S5).

The comparison between GAMM 03 and a respective null model without the parametric and nonparametric effects of condition revealed that condition significantly improved the model fit (Chi-square test of ML scores: χ^2^(5) = 6.66, p = 0.021; GAMM03 had a lower AIC: ΔAIC 19.35). The model summary showed a significant change of the pupil size over time in the Reversed condition (F(11.42, 13.56) = 2.57, p = 0.001) but not the Normal condition (F(1.01, 1.01) = 1.14, p = 0.285). The difference curve revealed no significant differences in pupil size between the Normal and Reversed condition (Figure 3D; for the model estimates and partial and summed effects see Table S6 and Figure S3).

### Experiment 2: Speed changes

**Figure 4:**
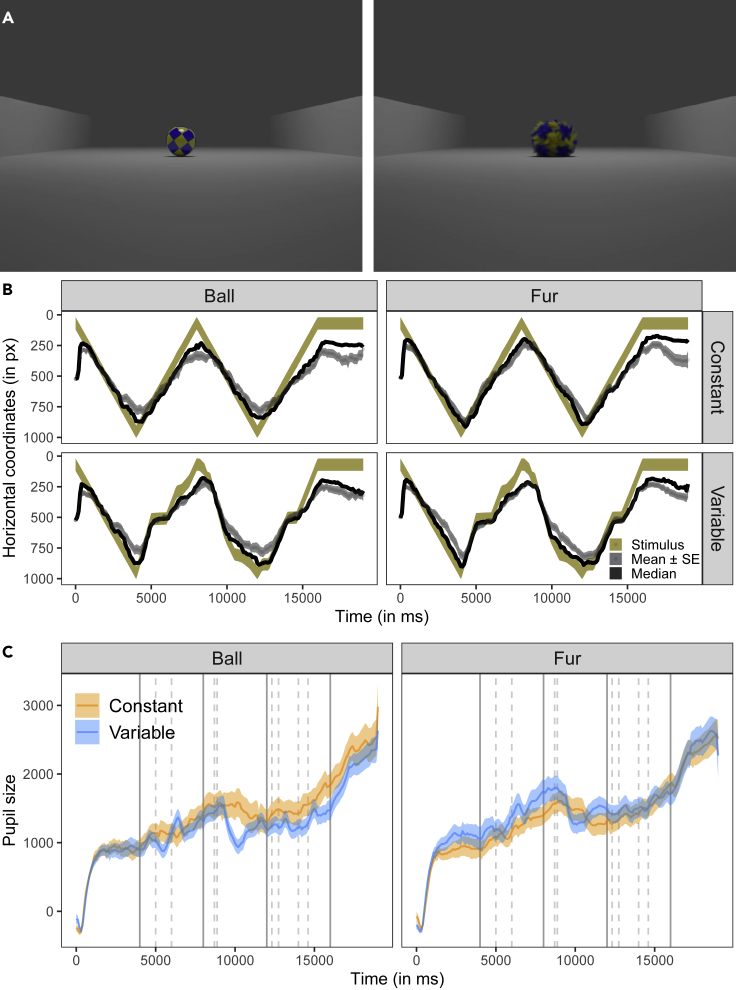
Screenshots and data visualizations of Experiment 2 (A) Screenshots of the videos (at 2.0 s; left: Ball; right: Fur ball). (B) Time series plot showing the dogs’ median (black line) and mean horizontal gaze coordinates (±se, dotted line and the dark gray shaded area around it; in px) in the test trials. The shaded yellow area represents the moving stimulus. (C) Time series plot showing dogs' pupil size (in arbitrary units and baseline corrected). The orange and blue lines show the mean pupil size (±se, shaded area around the line) in the Constant and Variable condition. The continuous vertical lines show the times when stimulus changes direction; the dashed vertical lines delimit periods when the ball was not moving in the Variable condition. The left facets show the Ball and right facets the Fur ball conditions.

In Experiment 2, we presented the dogs with realistic 3D animations of a moving, blue-yellow patterned ball rolling back and forth twice along a horizontal trajectory in between two walls (see Figure 4A, Video S2). We manipulated the appearance (fur/no fur) and the kinematic properties (constant/variable speed) of the ball in a 2 × 2 within-subject design. The ball had either a smooth surface (Ball condition) or a surface covered by fur (Fur condition), and it moved either at a constant (Constant condition) or at a variable speed (Variable condition; i.e. sometimes slowing down or stopping and then accelerating again).

Video S2. Video stimuli presented in Experiment 2 with the gaze of a dog overlaid, related to STAR Methods

In all four conditions, the horizontal movements of the ball explained a substantial amount of variance in dogs’ horizontal eye movements (mean r^2^ ± se; Ball-Constant: 0.58 ± 0.02, Ball-Variable: 0.65 ± 0.01; Fur-Constant: 0.64 ± 0.02, Fur-Variable: 0.70 ± 0.02; Figure 4B). An IA analysis of the interest period at the end of the video (with the ball in its end position) provided no evidence that the dogs looked longer at the ball in any of the conditions. We fitted a beta GLMM for the proportion of dwell times into the IA around the ball’s end position and included the categorical predictors’ motion (constant), stimulus (ball, fur), and their interaction as well as trial number within condition as covariate. The interaction between motion and stimulus was not significant (χ^2^(1) = 2.29, p = 0.130). A reduced model without the interaction revealed that neither the motion (χ^2^(1) = 0.34, p = 0.558; Table S7) nor the stimulus (χ^2^(1) = 1.69, p = 0.193) had a significant effect on dogs’ looking times. However, their looking times decreased with increasing trial number within condition (χ^2^(1) = 5.36, p = 0.021). There were no significant differences in the average fixation duration, the fixation count, or the mean saccade amplitude across conditions (see Tables S8 and S9).

The comparison between GAMM 04 and a respective null model without the parametric and nonparametric effects of condition revealed that condition significantly improved the model fit (Chi-square test of ML scores: χ^2^(11) = 260.36, p < 0.001; GAMM04 had a lower AIC: ΔAIC 681.28). The model summary showed a significant change of the pupil size over time in the Variable conditions (Ball: F(16.65, 17.15) = 10.05, p < 0.001; Fur: F(17.19, 17.83) = 6.75, p < 0.001) but not the Constant conditions (Ball: F(10.49, 11.65) = 1.37, p = 0.216; Fur: F(9.93, 11.02) = 0.32, p = 0.975). The pupil size also significantly declined with increasing session number (t = −3.15, p = 0.002). The difference curve or model summary provided no evidence for significant differences in pupil size between the four conditions (for the model estimates and partial and summed effects see Table S10 and Figure S8).

We fitted another GAMM (GAMM 05) just for the period around the first speed change (first right-left passage of the ball). Condition again significantly improved the model fit (full-null comparison: χ^2^(11) = 47.66, p < 0.001; GAMM05 had a lower AIC: ΔAIC 137.81). The model summary provided evidence for a significant change of the pupil size over time in all conditions except the Ball-Constant condition (Ball-Constant: F(5.84, 7.15) = 0.55, p = 0.831; Ball-Variable: F(14.75, 16.61) = 4.81, p < 0.001; Fur-Constant: F(10.23, 12.26) = 1.72, p = 0.037; Fur-Variable: F(13.37, 15.29) = 5.15, p < 0.001; for the model estimates and partial and summed effects see Table S11 and Figure S9).

## Discussion

Our results provide evidence that certain motion cues can lead to changes in the dogs’ pupil size over time. In Experiment 1, we found with three different videos that the dogs’ pupil size was more variable over time when the object moved in a self-propelled way in the Reversed condition compared with the Normal condition. In Experiment 2, we found similar results but this time for speed changes of a self-propelled object: the dogs’ pupil size varied more over time when the stimulus moved with variable speed (sometimes stopping and starting to move again) than when it moved at a constant speed. In contrast, there were no consistent differences in looking times or average fixation durations across the different experiments and conditions.

Which properties of the videos might explain the variable pupil size in the test conditions? The same visual elements were depicted across conditions and were controlled for the current gaze position in our pupil size analysis. Therefore, the stimulus appearance can be ruled out (except for the fur/no-fur comparison in Experiment 2). However, motion-related cues differed across conditions, particularly the direction of the stimulus movements in relation to the depicted agent (Experiment 1) or its kinematic properties (Experiment 2). Such cues have been associated with the detection of animacy in humans (e.g., Di Giorgio et al., 2021; Premack, 1990; Schlottmann and Surian, 1999; Scholl and Tremoulet, 2000; Träuble et al., 2014) and other animals (Greggor et al., 2018; Mascalzoni et al., 2010; Rosa-Salva et al., 2016; Wisenden and Harter, 2001).

We found only limited evidence that the appearance of the self-propelled object (i.e. the fur) had an effect on the dogs’ pupil size. Only when focusing on the first stop-start event within each trial of Experiment 2, we found that the pupil size also changed for the fur ball moving at a constant speed (unlike the smooth ball moving at constant speed). It is possible that dogs’ experience with furry (yet inanimate) toys explains why they did not show a stronger response to this manipulation. What might explain the difference between the fur-constant and ball-constant conditions, other than the dogs recognizing the fur as a property of animate beings? The rotation speed was identical between these two conditions. What differed, apart from the texture, was the size of the stimuli: to ensure that the rotation speed was identical, we added the fur to a same-sized ball, which resulted in a slightly larger stimulus. In addition, the animated fur moved when the ball changed the direction and speed. This served to highlight of the properties of the fur. However, we deem it unlikely that the stimulus size or the dynamic properties of the fur alone are responsible for the observed pupil size effect, given its similarity to the effects found for other animacy cues (speed changes and self-propulsion).

Increased pupil size is often interpreted as evidence for increased arousal (or mental load) when other effects such as luminosity can be excluded (Mathôt, 2018), an effect that has also been documented for dogs (Karl et al., 2020b; Somppi et al., 2017; Völter and Huber, 2021a). However, in the current study no clear pattern emerged with respect to overall pupil size differences across conditions. What differed consistently across the two studies was the trajectory of the pupil size over time, which was more variable in the conditions involving animacy cues. Therefore, the processing of such motion cues seems to have a different effect on dogs’ pupil size response compared with their reaction to physically impossible events (Völter and Huber, 2021a) and human emotional expressions (Karl et al., 2020b; Somppi et al., 2017). Expectancy violations and human emotional expressions might lead to increased arousal, which might be reflected in a slower and more pronounced pupil dilation response. The motion cues in the current study, in contrast, might be related to an orienting response, which also elicits a pupil dilation response in humans. This orienting-related pupil dilation response in humans is brief and elicited rapidly after attention has been captured (Mathôt, 2018), matching the pattern found in the current study. In general, the pupil response affects the visual sensitivity and acuity: sensitivity is increased when the pupils are dilated, whereas acuity is highest when the pupils are constricted (Campbell and Gregory, 1960; Mathôt and Van der Stigchel, 2015). Dogs’ variable pupil size as part of the orienting response to motion cues might allow them to efficiently balance visual acuity and sensitivity, which might be beneficial for detecting and tracking prey or conspecifics.

One might argue that the videos presented in the Reversed condition of Experiment 1 violated physical principles (i.e. the principle of support and gravity) but in a previous study we found no evidence that dogs formed expectations about support events in the context of screen-based stimuli (Völter and Huber, 2021a). Therefore, it appears unlikely that a violation of gravity-related expectations alone explained the results found in Experiment 1. Nevertheless, expectations about the physical environment and animacy perception might be linked as proposed by the energy violation hypothesis (Dittrich and Lea, 1994; Scholl and Tremoulet, 2000; Szego and Rutherford, 2008). This hypothesis predicts that motion cues suggestive of a hidden energy source such as speed and direction changes and movements that defy gravity in the absence of external causes lead to a perception of animacy. Our results indicate that such motion cues lead to an orienting response in dogs.

Previous studies with dogs already found some evidence that they are sensitive to biological motion (Eatherington et al., 2019, 2021; Ishikawa et al., 2018; Kovács et al., 2016), direction changes to avoid collisions with an obstacle (Tauzin et al., 2017), and chasing-like movement patterns (Abdai et al., 2017a, 2017b, 2020). Our previous study on contact causality further suggested that dogs discriminate launching events in which the movement of a ball stimulus resulted from a collision event from control events in which a ball started moving without any obvious external cause (Völter and Huber, 2021a). In line with these findings, the current study indicates that the dogs react to self-propulsion and speed changes. Their variable pupil response hints to an orienting response elicited by these cues. Whether the orienting response reflects a specific response to animate beings or is driven by an unspecific surprise reaction elicited by these cues remains an open question. Future studies could contrast surprising situations with and without animacy cues to clarify the extent to which this response is specific to animacy perception.

In humans, pupil size effects, to our knowledge, have not been examined in the context of animacy cues. However, studies have linked pupil dilation to motion perception. For example, there is evidence for pupil changes when participants perceived a change in direction of ambiguously moving stimuli (Hupé et al., 2009) and even when participants watched static images that merely implied motion (e.g., figurative paintings depicting moving objects; Castellotti et al., 2021). Our study raises the possibility that such links between motion processing and pupil size effects might be wide-spread across mammals. Future studies with predatory species (such as cats for which there is anecdotal evidence suggesting that they exhibit a marked pupil dilation in response to unfamiliar conspecifics in their home territory, familiar toy objects, and food; see Hess and Polt, 1960) and nonpredatory species are required to examine the extent to which such pupil dilation effects are phylogenetically conserved.

In sum, our findings suggest that dogs process objects that move in a self-propelled manner differently than objects that only move passively, for instance, when being released by an agent. Their sensitivity to self-propulsion was particularly evident by changes in their pupil size over time. We found a similar pupil size effect when comparing constant and variable movements of a self-propelled object and to some extent also when comparing a moving object with fur with one without fur. The current results support the notion that pupillometry might provide a sensitive way to study dogs’ information processing also when compared with looking time measures that for the most part did not reveal a clear pattern in this study. Together, these findings suggest that different motion cues that are useful for the detection of animate beings including self-propulsion and speed changes lead to an orienting response as reflected by their pupil response.

### Limitations of the study

The current study tested the dogs’ pupil size response to dynamic stimuli presented on a screen. Although there is research suggesting that dogs recognize stimuli on a screen (e.g., Autier-Dérian et al., 2013; Müller et al., 2015; Pongrácz et al., 2003; Range et al., 2008; Téglás et al., 2012), it remains unclear whether dogs’ pupil response would look the same with real-life stimuli. Future research with mobile eye-tracking and real-life demonstrations could help to clarify this issue. Second, the within-subject design introduces the risk of carry-over effects. Indeed, we found some evidence for an order effect in Experiment 2: the dogs’ pupil size decreased across sessions. Given that we had counterbalanced the order of conditions across subjects, we could account for these effects in the analysis. Testing the same individuals in multiple experiments, however, also bears some risk of carry-over effects across experiments even though we used different stimuli across experiments. Third, it remains an open question to what extent the observed pupil size response to self-propulsion and speed variability is specific to such animacy cues or whether it reflects a rather unspecific surprise response. Future research comparing potentially surprising events with and without animacy cues will help to clarify the specificity of the dogs’ pupil response. Relatedly, our study does not indicate whether self-propulsion and speed variability are independent cues for animacy cues. In Experiment 1, the objects moved with variable speed in both conditions. Adding conditions in which an object moving at constant speed is either shown in a normal or reversed sequence would show whether self-propulsion alone might be sufficient to elicit a response. Finally, our results do not show that the dogs perceived the stimulus in the test conditions *as* animate but they suggest that certain motion cues (typically referred to as animacy cues; e.g., Scholl and Tremoulet, 2000) elicit an orienting response in dogs.

## STAR★Methods

### Key resources table

**Table:** 

REAGENT or RESOURCE	SOURCE	IDENTIFIER
**Deposited data**		

Raw data (sample reports)	Zenodo repository	https://doi.org/10.5281/zenodo.6805817
Interest area reports	Zenodo repository	https://doi.org/10.5281/zenodo.6805817
Trial reports	Zenodo repository	https://doi.org/10.5281/zenodo.6805817
Video stimuli	Zenodo repository	https://doi.org/10.5281/zenodo.6805817

**Experimental model: Organisms/strains**		

Pet dogs (*Canis familiaris*)	private owners	N/A

**Software and algorithms**		

R scripts of data analysis	Zenodo repository	https://doi.org/10.5281/zenodo.6805817

**Other**		

Videos S1 and S2	Manuscript (supplemental information)	Videos S1 and S2

### Resource availability

#### Lead contact

Further information and requests for resources should be directed to and will be fulfilled by the Lead Contact, Christoph J. Völter (christoph.voelter@vetmeduni.ac.at).

#### Materials availability

The video stimuli used in the presented experiments have been deposited at Zenodo and are publicly available as of the date of publication. DOIs are listed in the key resources table.

### Experimental model and subject details

In Experiment 1, we tested 14 pet dogs (*Canis familiaris*; 5 border collies, 5 mixed breeds, 2 Labrador Retrievers, 1 collie, and 1 Australian Shepherd; mean age: 31.8 months, range: 14–80 months; 8 females, 6 males). In Experiment 2, we tested 17 dogs (including the same individuals as in Experiment 1; 5 border collies, 5 mixed breeds, 2 Labrador Retrievers, 1 collie, 1 Flat Coated Retriever, 1 Small Münsterländer, and 2 Australian Shepherd; mean age: 36.5 months, range: 14–81 months; 9 females, 8 males). All dogs that participated in this study were chin-rest trained (Karl et al., 2020a). The 14 dogs that participated in both experiments had previously also participated in previous eye-tracking studies on expectancy violations of physical regularities (Völter and Huber, 2021a, 2021b).

The study was discussed and approved by the institutional ethics and animal welfare committee in accordance with GSP guidelines and national legislation (approval number: ETK-066/03/2020). Written consent to participate in the study was obtained by the dogs’ owners.

### Method details

#### Stimuli

In Experiment 1, we presented the dogs with three different videos (frame rate: 100 fps): the cube, ball, and rope video. The cube video (4 s; see Figure 1A) showed an actor (shown from the side) holding a foam cube in his outstretched hand and then dropping it. When the cube made contact with the ground, it bounced off twice before coming to a rest. The actor followed the cube movements with his gaze. In the ball video (3.5 s; see Figure 2A), a human hand is shown (from the side) holding a blue ball at the upper end of a slanted surface. The hand released the ball and the ball rolled down the surface. At the lower end of the slanted surface, the ball hit a vertical wall from which it bounced off twice before coming to a rest. In the rope video (2 s; see Figure 3A), a human hand was shown holding a short piece of a multicolored rope. The hand then released the rope and it fell down to the ground. Depending on the condition, the dogs watched forward or reverse playing versions of these videos. At the end of the video, the last frame of the video was shown for 1 s.

We matched the conditions based on time (from the start of the video). Matching conditions based on the picture (e.g., by re-aligning the pupil size data) would have introduced another confounding factor: changes in the pupil size over time. We sometimes observe a steady increase in the pupil size over time (the reasons are not entirely clear, e.g., increased arousal with increasing video duration or expectation of a reward that was provided at the end of each run). Therefore, had we matched the videos by the picture, the effect of time would have confounded the comparison between conditions. In Experiment 2, we presented the dogs with four different videos (16 s; 100fps; created in Blender 2.8). In all videos, a ball moved along the same horizontal motion path. The scene depicts just a grey surface with grey vertical walls on each side. At the beginning of the videos the ball is located on the left edge of the scene. The ball then rolled from left to right and reversed the direction when making contact with the right wall. This back-and-forth sequence is repeated once (complete rolling sequence: left-right-left-right). Each passage from one side to the other took 4 s. The balls were blue and yellow patterned. Depending on the video, the ball had either a smooth surface (Ball condition) or a surface covered by fur (Fur condition). Depending on the video, the ball either moved with constant speed (constant condition) or with variable speed (variable condition). The last frame of the video was shown for 4 s (total trial duration: 20 s).

#### Apparatus

We tracked the dogs’ eye movements by means of the EyeLink1000 eye-tracking system (SR Research, Canada) at a rate of 1000 Hz. We used an adjustable chin rest to facilitate the maintenance of a stable head position during video presentation (Karl et al., 2020a). We presented the videos on a 24-inch LCD monitor (resolution: 1024 × 768; refresh rate: 100 Hz) at a distance of 70 cm from the dogs’ eyes. The video area subtended visual angles of 31.89 (horizontal) and 24.19 (vertical) degrees. In Experiment 1, the moving stimuli had a diameter of ca. 80 px (cube), 110 px (ball), and 450–550px (rope; exact values changing over the course of the video) subtending a visual angle of 2.56, 3.52 and 14.31–17.45 degrees, respectively. In Experiment 2, the ball had a diameter of ca. 85 px (smooth surface; visual angle: 2.72) and 125 × 95 px (h x v; furry surface; visual angle: 4 × 3.04). The fur ball was slightly larger because the fur was added to a ball of the same size as in the other condition. In that way, we kept the angular velocity constant across conditions. We used the centroid pupil shape model to fit the pupil and determine the pupil position. The tracking rate was high (Experiment 1: 99.3% tracked, after removal of blink artifacts: 98.2%).

#### Design and procedure

In Experiment 1, we presented the dogs with three different video recordings, one for each sub-experiment (Cube, Ball, and Rope video). In each sub-experiment, the dogs participated in two (within-subject) conditions: Normal and Reversed. In the Normal condition, the video was played forward; in the Reversed condition, it was played in reverse direction. For each sub-experiment and condition, the dogs watched the same video four times in a row. The order of condition for each sub-experiment was counterbalanced across dogs. We pseudo-randomly assigned the dogs to the order groups and counterbalanced the groups as much as possible with respect to age, sex, and breed. Participation in the study required two visits (sessions) in the lab. In each session, the dogs were presented with one condition of each sub-experiment. The dogs were presented with the two conditions of each sub-experiment on separate days (minimum 5 days between sessions). The sub-experiments were presented in a fixed order (cube → rope → ball). There was a short break in between the sub-experiments. When the dog left the chin rest in between sub-experiments we conducted another calibration before starting the next sub-experiment.

In Experiment 2, we administered a 2 × 2 within-subject design in which we manipulated the appearance of the moving stimulus (smooth surface or fur) and the movement properties (constant or variable speed). The order of conditions was counterbalanced across dogs; the assignment to the order groups was pseudo-random (see above). We conducted four sessions per dog. The dogs were presented with the four conditions on separate days (minimum 2 days between sessions). In each session, the dogs watched the same video three times in a row.

In each session, the dogs first completed a 5-point calibration with animated calibration targets (32–64 px) subtending visual angles of 1.02–2.05° depending on the used target stimulus. Following the calibration, a central fixation target was presented (Exp. 1: a white pulsating circle; max diameter: 95 px; visual angle: 3.04°; Exp. 2: the calibration target); the video started once the dogs fixated the target for 50 msec.

### Quantification and statistical analysis

In order to analyze to what extent the vertical/horizontal coordinates of the moving stimulus (cube, ball, or rope) predicted variance in dogs’ vertical (Exp. 1: Cube and Rope) or horizontal (Exp. 1: Ball, Exp. 2) looking behavior, we calculated the proportion of variance (r^2^) values for the period between 500 ms after the onset of the video and the end of the video. The main stimulus movement occurred along the vertical axis in the Cube and Rope videos but along the horizontal axis in the Ball video and in the videos shown in Experiment 2. We used the main movement direction for the movement tracking analysis. We determined the location of the stimuli by means of the dynamic interest area (IA) recording function in EyeLink Data Viewer. We fitted a linear model for each subject with the dogs’ vertical or horizontal gaze positions as the response variable and the y or x-coordinates of the stimulus center as the predictor variable. Finally, we obtained the proportion of variance (r^2^) explained by the stimulus coordinates from these models.

For the dwell time analysis, we analyzed the interest period at the end of the video when the last frame of the video was shown for 1 s (Exp. 1)/4 s (Exp. 2). We defined IA around the end positions of the moving object (w x h: Exp. 1: cube 130 × 130 px; ball 200 × 200 px; rope: 500 × 300 px; Exp. 2: 230 × 300 px). In the Cube video of Experiment 1, we also analyzed the dogs’ dwell times to the actor’s head (head IA: w x h: 250 × 160 px) and the number of visited IAs (upper and lower cube position and actor’s head, including revisits). In Experiment 1, we compared the dwell time to the respective end positions between conditions using two-tailed, paired-samples t-tests. In Experiment 2, we fitted a generalized linear mixed model (GLMM) with beta error distribution for the proportion dwell time in the end position IA (using R package glmmTMB; Brooks et al., 2017). We included the predictor variables motion (constant, variable), stimulus (ball, fur), their interaction, and trial number within condition (1–3) as predictor variables, subject ID as random intercept, and the random slopes of motion, stimulus, and trial number within subject ID. We checked for overdispersion, which was no issue (dispersion parameter: 0.99). We also checked for collinearity, which was no issue either (maximum Variance Inflation Factor: 1.0; Field, 2005).

The pupil size data were preprocessed as follows: we first plotted individual time series to detect potential blink or measurement artifacts (see below). We excluded samples 100ms before and after detected blink events, applied a linear interpolation, and conducted a subtractive baseline correction (Mathôt et al., 2018) by using the first 200 ms (Exp. 1, i.e. before the stimuli started moving) and the first 1000 ms (Exp. 2, i.e. before any speed changes were introduced). Finally, we sampled the data down to 10 Hz to mitigate potential autocorrelation issues. Down sampling was achieved by selecting the median pupil size values within 100 ms bins.

Two dogs did not complete the last trial of the Reversed condition in the Ball sub-experiment, we therefore also excluded corresponding trials of the Normal condition of these two dogs from the pupil size analysis. One further trial was excluded due to a likely measurement artefact (misdetection of the pupil): In the second trial of the Reversed condition in the cube experiment, one dog had pupil sizes at the beginning of the video more than twice the magnitude of the values for any other dog or of the same dog in any other trial. We removed this trial and the corresponding trial of the Normal condition from the data analysis.

We analyzed the preprocessed pupil size data by fitting a generalised additive mixed model (GAMM) with Gaussian error structure (Sóskuthy, 2017; van Rij et al., 2019). We analyzed the entire remaining trial from the end of the baseline period. In Experiment 2, we fitted a further GAMM for a shorter interest period (4 s) that spanned the first right-left movement of the stimulus (including the first speed changes). We fitted the GAMM in R using the function 'bam' of package 'mgcv' (Wood, 2011) and package 'itsadug' (van Rij et al., 2020) for visualization. We used smoothing parameter selection method ‘ML’. We included condition and an order term (Exp. 1: order of condition; Exp. 2: session number) as parametric terms, the non-parametric regression lines for time and for the two levels of condition over time (with the upper limit for the number of knots set to 20), and the non-linear interaction between X and Y gaze positions (because previous research has shown that the gaze position can affect the pupil size (Mathôt et al., 2018; van Rij et al., 2019)). Additionally, we included random factor smooths for each individual time series trajectory (i.e. for each subject and test trial) and for each subject to improve the model fit and to account for autocorrelation in the data (Sóskuthy, 2017; van Rij et al., 2019).

We evaluated the model fit of the GAMM by inspecting visualizations of correlations between the residuals and the lagged residuals, a QQ-plot of residuals as well as the residuals against the fitted values (using the functions 'gam.check' of package 'mgcv' and 'acf' of package 'stats'). We found no obvious violation of the model assumptions. We evaluated the significance of condition on the pupil size by comparing the full model to a reduce model excluding both the parametric and smooth terms of condition using a Chi-square test of ML scores (using the function compareML of R package ‘itsadug’), evaluating the model summary, and inspecting the estimates of the differences between the conditions (using the function plot_diff of R package ‘itsadug’) (van Rij et al., 2020).

## Data Availability

The data and R scripts associated with this manuscript have been deposited at Zenodo and are publicly available as of the date of publication. DOIs are listed in the key resources table. Any additional information required to reanalyze the data reported in this work paper is available from the Lead Contact upon request.
