# Supplementary material for: Pupil size changes reveal dogs’ sensitivity to motion cues
Source: iScience. 2022 Jul 20;25(9):104801. doi: 10.1016/j.isci.2022.104801 (PMC9424576; doi:10.1016/j.isci.2022.104801)
Supplement: Document S1. Figures S1–S9 and Tables S1–S11 [file mmc1.pdf]

iScience, Volume *25*

# Supplemental information

# Pupil size changes reveal dogs' sensitivity to motion cues

**Christoph J. Völter and Ludwig Huber**

*Table S1 Comparison of the dogs' gaze behavior across the two test conditions of the cube experiment, related to Figure 1*

| **Cube** | condition | mean | se | t | df | p | lower CI | upper CI |
|---|---|---|---|---|---|---|---|---|
| Fixation duration | normal | 590.42 | 18.81 | 0.83 | 13 | 0.42 | -75.90 | 170.81 |
| | reversed | 542.97 | 12.85 | | | | | |
| Fixation count | normal | 9.50 | 0.23 | -0.31 | 13 | 0.76 | -1.69 | 1.26 |
| | reversed | 9.71 | 0.21 | | | | | |
| Saccade amplitude | normal | 5.96 | 0.19 | 2.33 | 13 | 0.04 | 0.11 | 2.90 |
| | reversed | 4.45 | 0.08 | | | | | |
| Saccade count | normal | 8.61 | 0.22 | -0.26 | 13 | 0.80 | -1.67 | 1.32 |
| | reversed | 8.79 | 0.21 | | | | | |

Notes: Fixation duration in msec. Saccade amplitude in degrees of visual angle. T-tests refer to exact, paired-samples t-tests.

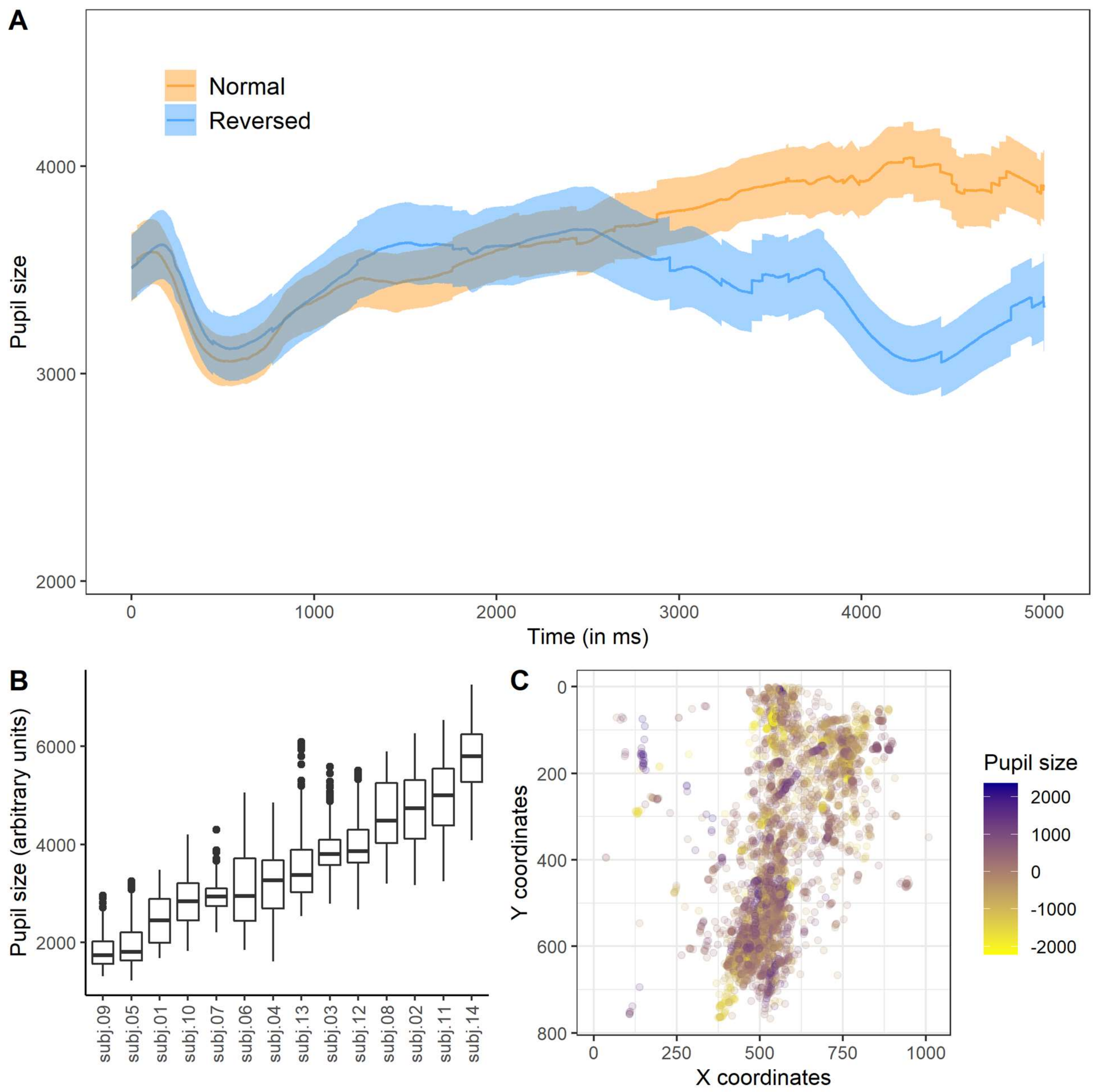


*Figure S1.* Experiment 1 (Cube video): Plots of the pupil size data as a function of time, subjects, and screen coordinates; related to Figure 1. (A) Mean pupil size (±SE; not baseline corrected, without blink artefacts) over time across the two conditions; (B) Pupil size variability (raw data without blink artefacts) across subjects; (C) Pupil sizes (preprocessed and baseline corrected) as a function of gaze position (x and y screen coordinates).

*Table S2 Results of GAMM 01 (Experiment 1: Cube); related to Figure 1*

| Parametric coefficients: | | | | |
|---|---|---|---|---|
| | Estimate | SE | t | p-value |
| (Intercept) | -48.79 | 138.77 | -0.35 | 0.725 |
| Condition | -215.83 | 107.33 | -2.01 | 0.044 |
| Order of condition | 265.38 | 196.37 | 1.35 | 0.177 |
| Smooth terms | | | | |
| | edf | Ref.df | F | p-value |
| s(Time) | 14.06 | 14.81 | 7.98 | <0.001 |
| s(Time):ConditionNormal | 4.85 | 5.34 | 0.44 | 0.841 |
| s(Time):ConditionReversed | 15.49 | 16.48 | 5.28 | <0.001 |
| s(Xgaze,Ygaze) | 26.43 | 28.32 | 19.92 | <0.001 |
| s(Time,Event) | 842.67 | 987.00 | 132.65 | <0.001 |
| s(Time,Subject) | 73.01 | 124.00 | 1.59 | <0.001 |

Notes: Reference category of condition: Normal, order of condition: Normal-first

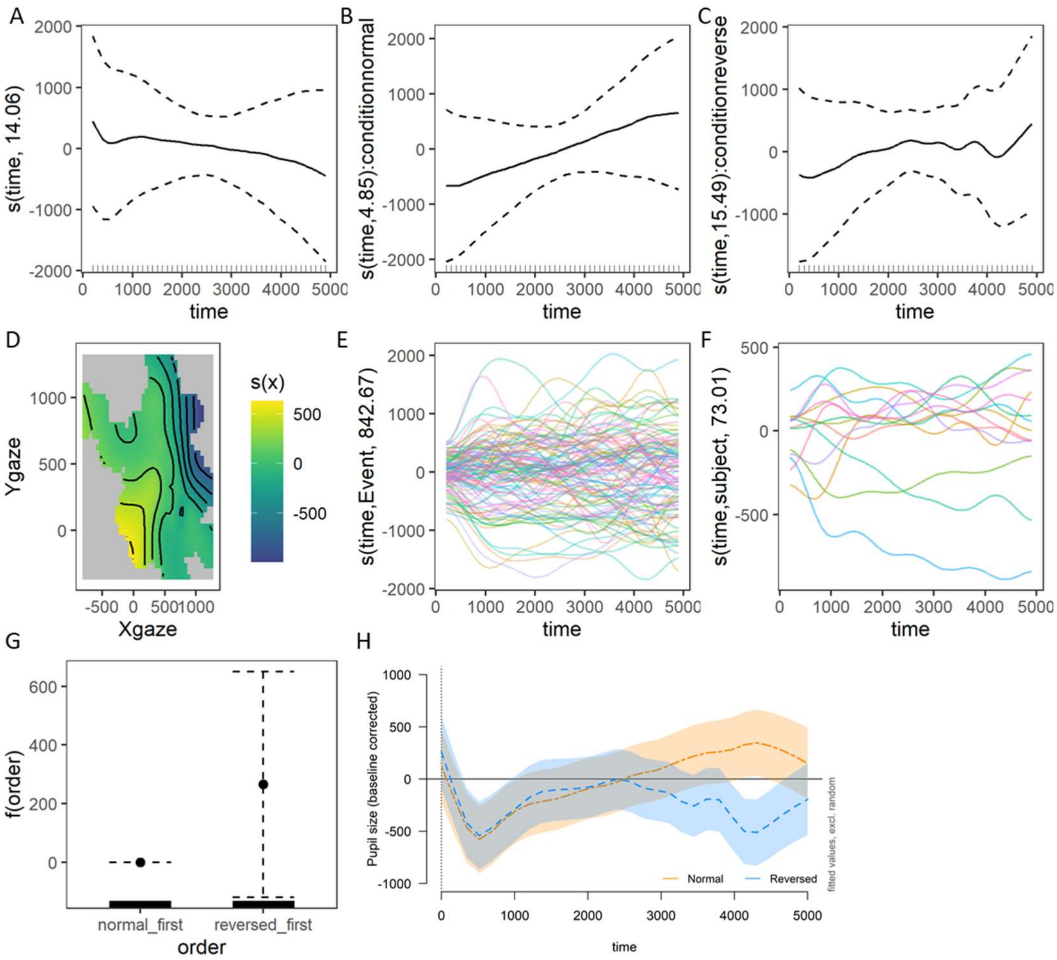


*Figure S2.* Plots of the partial and summed effects of GAMM 01 (Experiment 1: Cube) ; related to Figure 1. All parametric and smooth components are plotted. A: smoothing term of time; B: Smoothing term of the Normal condition over time; C: Smoothing term of the Reversed condition over time; D: Heatmap with overlaid contours of the 2D smooth of the X and Y gaze coordinates; E: random factor smooths for each trial and individual (N=110); F: random factor smooths for each individual (N=14) G: Parametric effect of order of condition (normal first, reversed first); H: summed effects for both conditions (random effects set to 0). The partial effects plots have been created using the function `getViz` of the package mgcViz (Fasiolo et al., 2018). The summed effects plot has been created using the function `plot_smooth` of the package itsadug (van Rij et al., 2020).

*Table S3 Comparison of the dogs' gaze behavior across the two test conditions of the ball experiment; related to Figure 2.*

| **Cube** | condition | mean | se | t | df | p | lower CI | upper CI |
|---|---|---|---|---|---|---|---|---|
| Fixation duration | normal | 572.92 | 11.23 | -0.44 | 13 | 0.66 | -97.12 | 63.95 |
| | reversed | 589.50 | 8.63 | | | | | |
| Fixation count | normal | 7.89 | 0.13 | 0.59 | 13 | 0.56 | -0.74 | 1.30 |
| | reversed | 7.61 | 0.11 | | | | | |
| Saccade amplitude | normal | 7.45 | 0.15 | -0.75 | 13 | 0.46 | -1.77 | 0.85 |
| | reversed | 7.91 | 0.12 | | | | | |
| Saccade count | normal | 7.04 | 0.12 | 0.81 | 13 | 0.44 | -0.61 | 1.34 |
| | reversed | 6.67 | 0.11 | | | | | |

Notes: Fixation duration in msec. Saccade amplitude in degrees of visual angle. T-tests refer to exact, paired-samples t-tests.

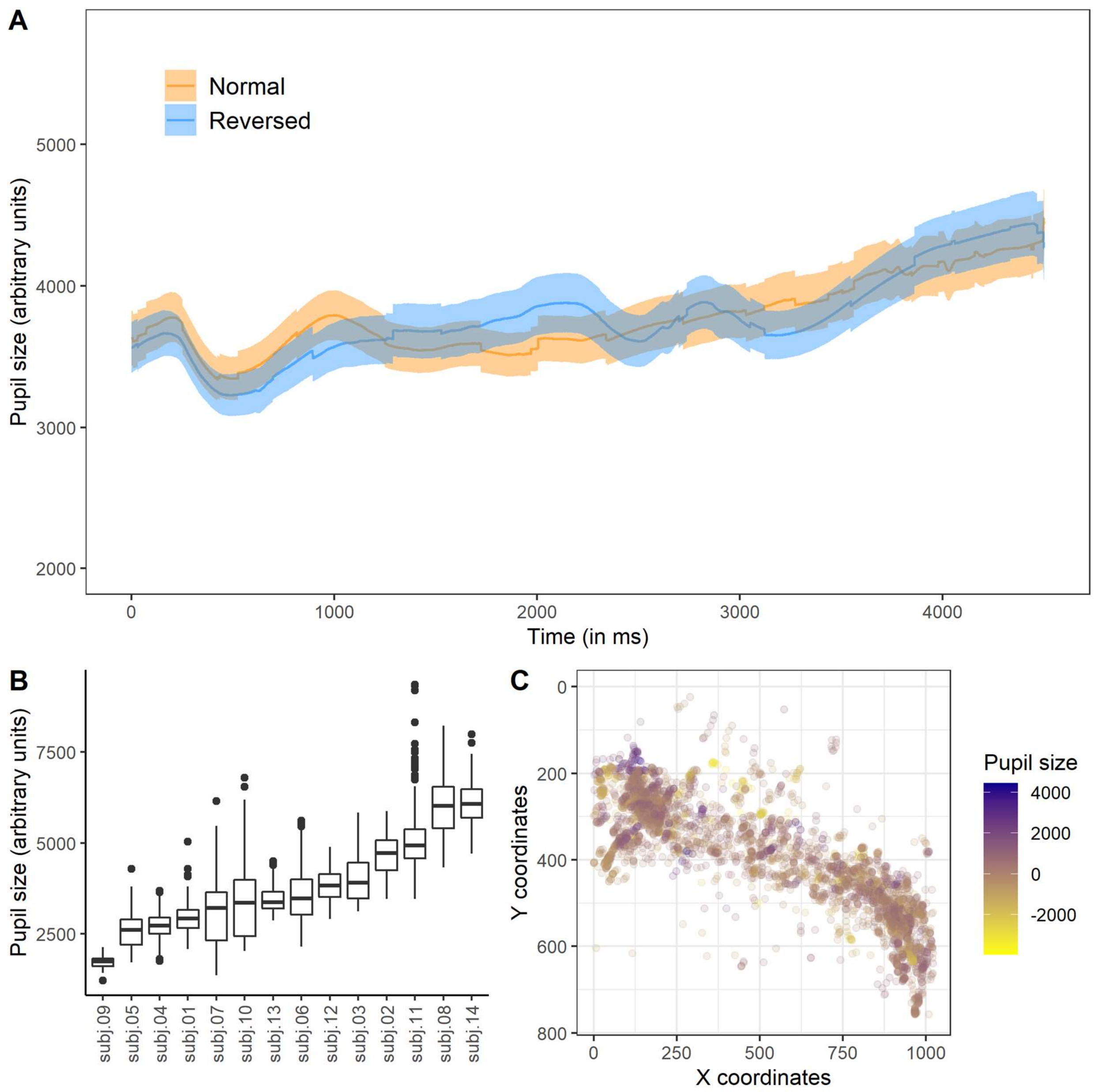


*Figure S3.* Experiment 1 (Ball video): Plots of the pupil size data as a function of time, subjects, and screen coordinates; related to Figure 2. (A) Mean pupil size (±SE; not baseline corrected, without blink artefacts) over time across the two conditions; (B) Pupil size variability (raw data without blink artefacts) across subjects; (C) Pupil sizes (preprocessed and baseline corrected) as a function of gaze position (x and y screen coordinates).

*Table S4 Results of GAMM 02 (Experiment 1: Ball); related to Figure 2*

| Parametric coefficients: | | | | |
|---|---|---|---|---|
| | Estimate | SE | t | p-value |
| (Intercept) | -228.42 | 259.78 | -0.88 | 0.379 |
| Condition | 59.61 | 151.29 | 0.39 | 0.694 |
| Order of condition | 202.81 | 151.21 | 1.34 | 0.180 |
| Smooth terms | | | | |
| | edf | Ref.df | F | p-value |
| s(Time) | 13.998 | 14.52 | 4.018 | <0.001 |
| s(Time):ConditionNormal | 9.319 | 10.13 | 0.376 | 0.8789 |
| s(Time):ConditionReversed | 10.804 | 11.81 | 2.032 | 0.0402 |
| s(Xgaze,Ygaze) | 24.907 | 27.73 | 37.078 | <0.001 |
| s(Time,Event) | 848.116 | 987 | 176.491 | <0.001 |
| s(Time,Subject) | 73.369 | 124 | 1.618 | <0.001 |

Notes: Reference category of condition: Normal, order of condition: Normal-first

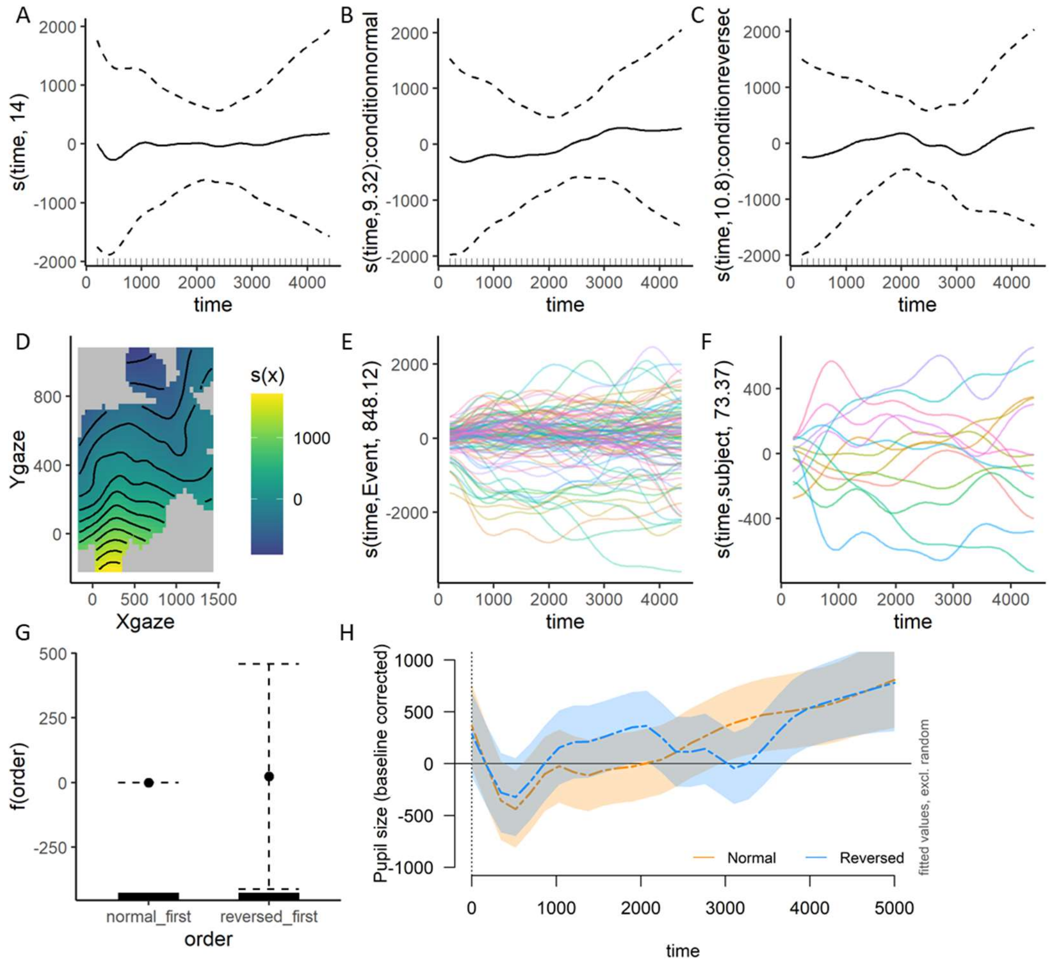


*Figure S4* Plots of the partial and summed effects of GAMM 02 (Experiment 1: Ball); related to Figure 2. All parametric and smooth components are plotted. A: smoothing term of time; B: Smoothing term of the Normal condition over time; C: Smoothing term of the Reversed condition over time; D: Heatmap with overlaid contours of the 2D smooth of the X and Y gaze coordinates; E: random factor smooths for each trial and individual (N=108); F: random factor smooths for each individual (N=14) G: Parametric effect of order of condition (normal first, reversed first); H: summed effects for both conditions (random effects set to 0). The partial effects plots have been created using the function `getViz` of the package mgcViz (Fasiolo et al., 2018). The summed effects plot has been created using the function `plot_smooth` of the package itsadug (van Rij et al., 2020).

*Table S5 Comparison of the dogs' gaze behavior across the two test conditions of the rope experiment; related to Figure 3.*

| **Cube** | condition | mean | se | t | df | p | lower CI | upper CI |
|---|---|---|---|---|---|---|---|---|
| Fixation duration | normal | 500.84 | 8.29 | -1.46 | 13 | 0.17 | -149.35 | 28.82 |
| | reversed | 561.10 | 10.48 | | | | | |
| Fixation count | normal | 6.04 | 0.12 | 1.31 | 13 | 0.21 | -0.34 | 1.37 |
| | reversed | 5.52 | 0.07 | | | | | |
| Saccade amplitude | normal | 6.73 | 0.12 | 0.10 | 13 | 0.92 | -1.36 | 1.50 |
| | reversed | 6.66 | 0.20 | | | | | |
| Saccade count | normal | 5.14 | 0.12 | 1.28 | 13 | 0.22 | -0.32 | 1.25 |
| | reversed | 4.68 | 0.08 | | | | | |

Notes: Fixation duration in msec. Saccade amplitude in degrees of visual angle. T-tests refer to exact, paired-samples t-tests.

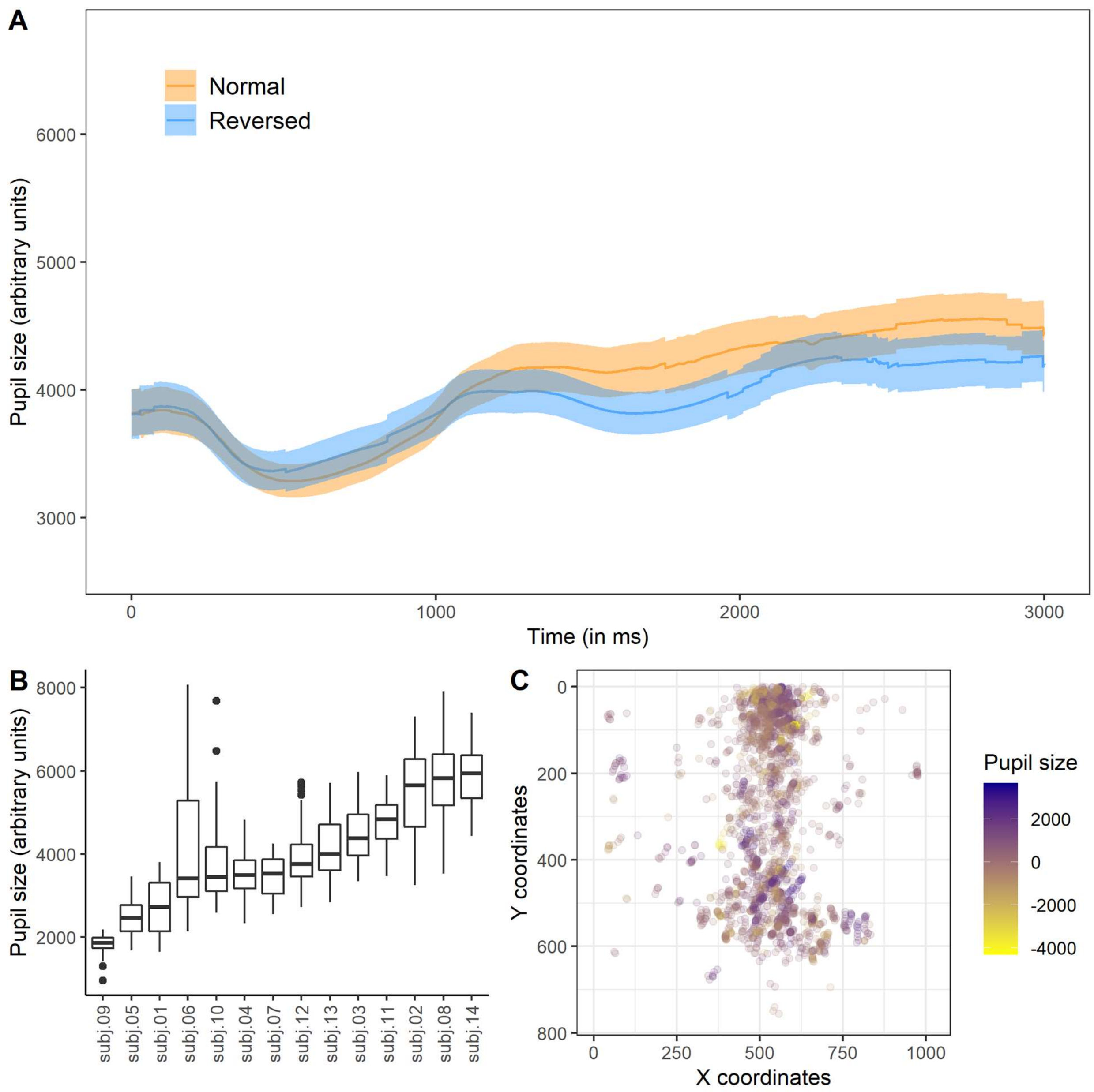


*Figure S5.* Experiment 1 (Rope video): Plots of the pupil size data as a function of time, subjects, and screen coordinates; related to Figure 3. (A) Mean pupil size (±SE; not baseline corrected, without blink artefacts) over time across the two conditions; (B) Pupil size variability (raw data without blink artefacts) across subjects; (C) Pupil sizes (preprocessed and baseline corrected) as a function of gaze position (x and y screen coordinates).

*Table S6 Results of GAMM 03 (Experiment 1: Rope); related to Figure 3.*

| Parametric coefficients: | | | | |
|---|---|---|---|---|
| | Estimate | SE | t | p-value |
| (Intercept) | 307.40 | 258.10 | 1.19 | 0.234 |
| Condition | -134.00 | 141.30 | -0.95 | 0.343 |
| Order of condition | -185.20 | 351.10 | -0.53 | 0.598 |
| Smooth terms | | | | |
| | edf | Ref.df | F | p-value |
| s(Time) | 15.93 | 17.04 | 26.14 | <0.001 |
| s(Time):ConditionNormal | 1.01 | 1.01 | 1.14 | 0.285 |
| s(Time):ConditionReversed | 11.42 | 13.56 | 2.57 | 0.001 |
| s(Xgaze,Ygaze) | 21.81 | 25.69 | 23.61 | <0.001 |
| s(Time,Event) | 851.27 | 1005.00 | 351.31 | <0.001 |
| s(Time,Subject) | 98.38 | 124.00 | 5.01 | <0.001 |

Notes: Reference category of condition: Normal, order of condition: Normal-first

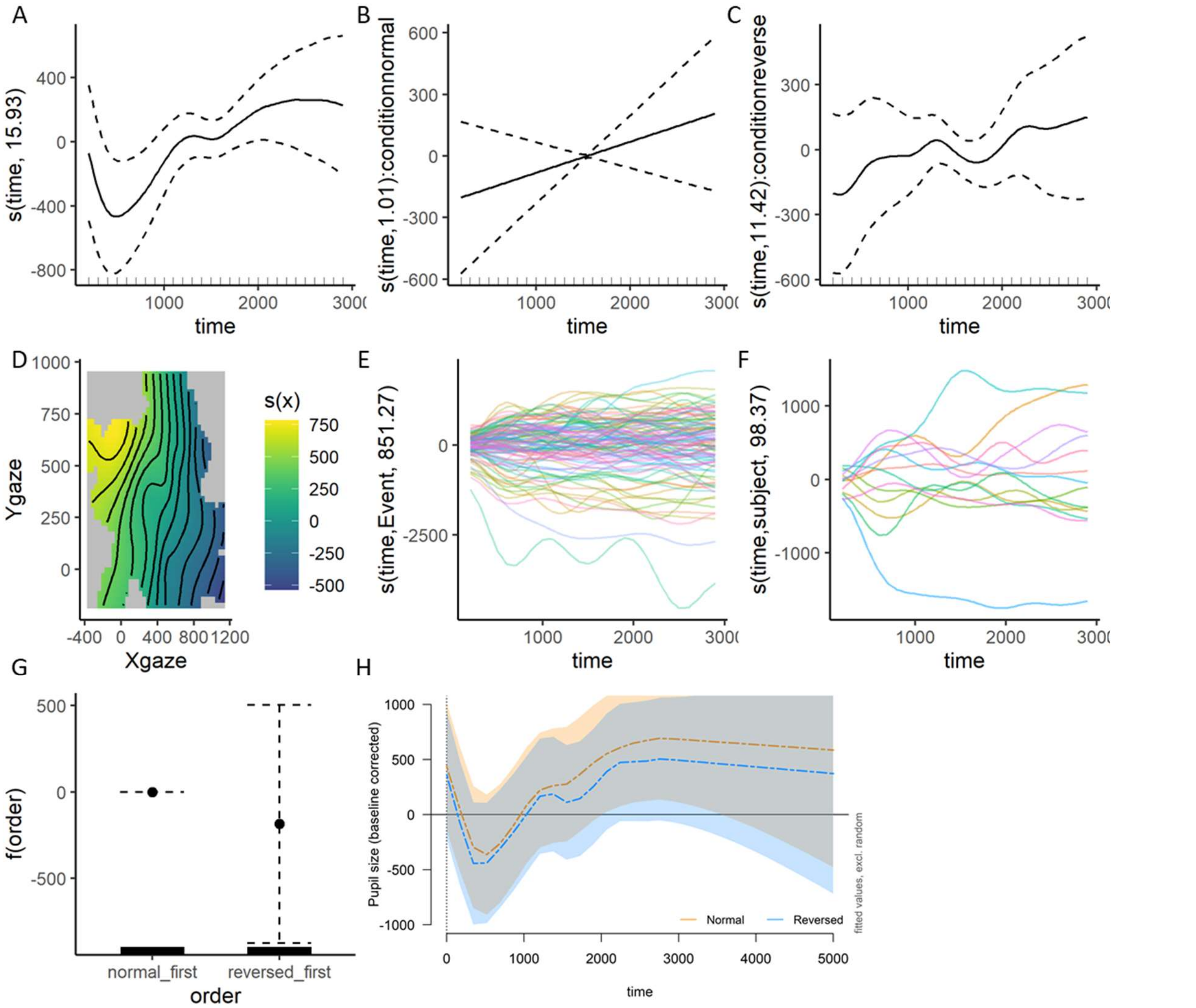


*Figure S6.* Plots of the partial and summed effects of GAMM 03 (Experiment 1: Rope); related to Figure 3. All parametric and smooth components are plotted. A: smoothing term of time; B: Smoothing term of the Normal condition over time; C: Smoothing term of the Reversed condition over time; D: Heatmap with overlaid contours of the 2D smooth of the X and Y gaze coordinates; E: random factor smooths for each trial and individual (N=112); F: random factor smooths for each individual (N=14) G: Parametric effect of order of condition (normal first, reversed first); H: summed effects for both conditions (random effects set to 0). The partial effects plots have been created using the function `getViz` of the package mgcViz (Fasiolo et al., 2018). The summed effects plot has been created using the function `plot_smooth` of the package itsadug (van Rij et al., 2020).

*Table S7 Results of GLMM with beta error distribution of the proportion looking time to the area of interest around the end position of the ball at the end of the video (Experiment 2); related to Figure 4.*

| | Estimate | SE | χ2 | df | p |
|---|---|---|---|---|---|
| (Intercept) | -0.72 | 0.23 | | | |
| Motion[1] | 0.10 | 0.18 | 0.34 | 1 | 0.558 |
| Stimulus[2] | 0.23 | 0.18 | 1.69 | 1 | 0.193 |
| Trial[3] | -0.21 | 0.09 | 5.36 | 1 | 0.021 |

Notes: Reference category: [1]constant; [2]variable; [3]Trial was z-transformed to a mean of 0 and an sd of 1.

*Table S8 Descriptive statistics concerning dogs' gaze behavior across the test conditions of Experiment 2; related to Figure 4.*

| | condition | mean | se |
|---|---|---|---|
| Fixation duration | B-C | 901.14 | 16.44 |
| | B-V | 992.41 | 19.74 |
| | F-C | 977.75 | 20.35 |
| | F-V | 903.72 | 19.74 |
| Fixation count | B-C | 21.84 | 0.36 |
| | B-V | 20.57 | 0.38 |
| | F-C | 20.73 | 0.32 |
| | F-V | 23.51 | 0.49 |
| Saccade amplitude | B-C | 5.82 | 0.12 |
| | B-V | 5.73 | 0.13 |
| | F-C | 5.80 | 0.10 |
| | F-V | 5.35 | 0.13 |
| Saccade count | B-C | 21.00 | 0.36 |
| | B-V | 19.69 | 0.38 |
| | F-C | 19.80 | 0.32 |
| | F-V | 22.61 | 0.49 |

Notes: Fixation duration in msec. Saccade amplitude in degrees of visual angle.

*Table S9 Comparison of the dogs' gaze behavior across the test conditions of Experiment 2; related to Figure 4.*

| | Comparison | t | df | p | lower CI | upper CI |
|---|---|---|---|---|---|---|
| Fixation duration | Motion | -0.23 | 16 | 0.824 | -89.36 | 72.12 |
| | Stimulus | 0.12 | 16 | 0.903 | -97.15 | 109.24 |
| Fixation count | Motion | -1.02 | 16 | 0.324 | -2.33 | 0.82 |
| | Stimulus | -0.85 | 16 | 0.406 | -3.18 | 1.36 |
| Saccade amplitude | Motion | 0.87 | 16 | 0.399 | -0.39 | 0.92 |
| | Stimulus | 0.63 | 16 | 0.538 | -0.48 | 0.88 |
| Saccade count | Motion | -0.99 | 16 | 0.335 | -2.34 | 0.85 |
| | Stimulus | -0.79 | 16 | 0.439 | -3.17 | 1.44 |

Notes: Motion refers to the constant-variable motion comparison; stimulus refers to the ball-fur comparison. Paired samples t-tests based on aggregated data are reported.

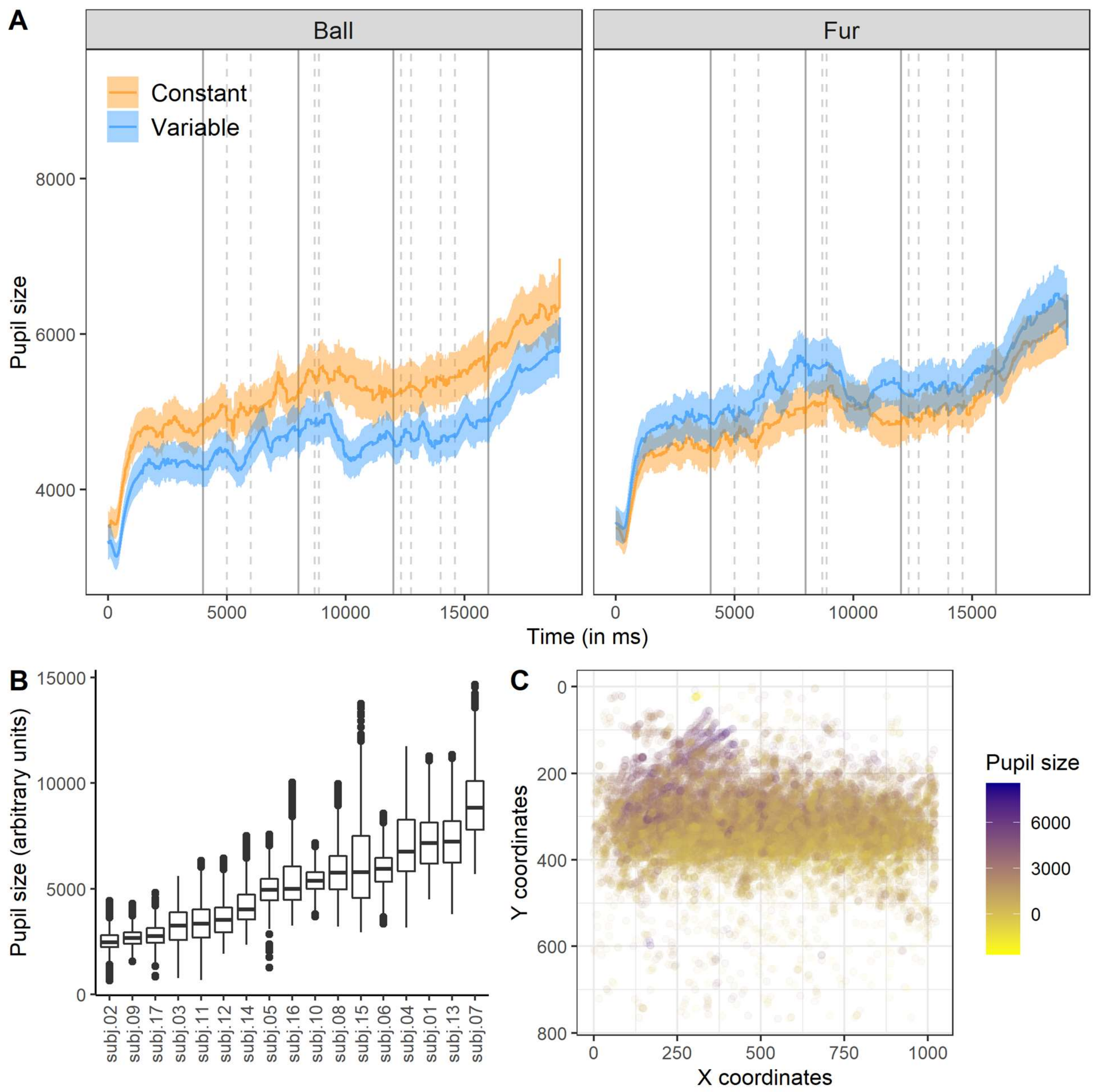


*Figure S7.* Experiment 2: Plots of the pupil size data as a function of time, subjects, and screen coordinates; related to Figure 4. (A) Mean pupil size (±SE; not baseline corrected, without blink artefacts) over time across the two movement conditions and the two stimulus conditions (left facet: ball without fur; right facet: fur ball). The continuous vertical lines show the times when stimulus changes direction; the dashed vertical lines delimit periods when the ball was not moving in the Variable condition. (B) Pupil size variability (raw data without blink artefacts) across subjects. (C) Pupil sizes (preprocessed and baseline corrected) as a function of gaze position (x and y screen coordinates).

*Table S10 Results of GAMM 04 (Experiment 2); related to Figure 4.*

| Parametric coefficients: | | | | |
|---|---|---|---|---|
| | Estimate | SE | t | p-value |
| (Intercept) | 1808.39 | 220.31 | 8.21 | <0.001 |
| ConditionBall-Variable | -213.40 | 143.83 | -1.48 | 0.138 |
| ConditionFur-Constant | -94.76 | 143.26 | -0.66 | 0.508 |
| ConditionFur-Variable | -8.01 | 143.91 | -0.06 | 0.956 |
| Session | -110.67 | 35.11 | -3.15 | 0.002 |
| Smooth terms | | | | |
| | edf | Ref.df | F | p-value |
| s(Time) | 14.44 | 15.01 | 4.23 | <0.001 |
| s(Time):ConditionBall-Constant | 10.49 | 11.65 | 1.37 | 0.216 |
| s(Time):ConditionBall-Variable | 16.65 | 17.15 | 10.05 | <0.001 |
| s(Time):ConditionFur-Constant | 9.93 | 11.02 | 0.32 | 0.975 |
| s(Time):ConditionFur-Variable | 17.19 | 17.83 | 6.75 | <0.001 |
| s(Xgaze,Ygaze) | 28.59 | 28.98 | 218.30 | <0.001 |
| s(Time,Event) | 1633.17 | 1831.00 | 92.04 | <0.001 |
| s(Time,Subject) | 97.98 | 152.00 | 3.48 | <0.001 |

Notes: Reference category of condition: Ball-Constant.

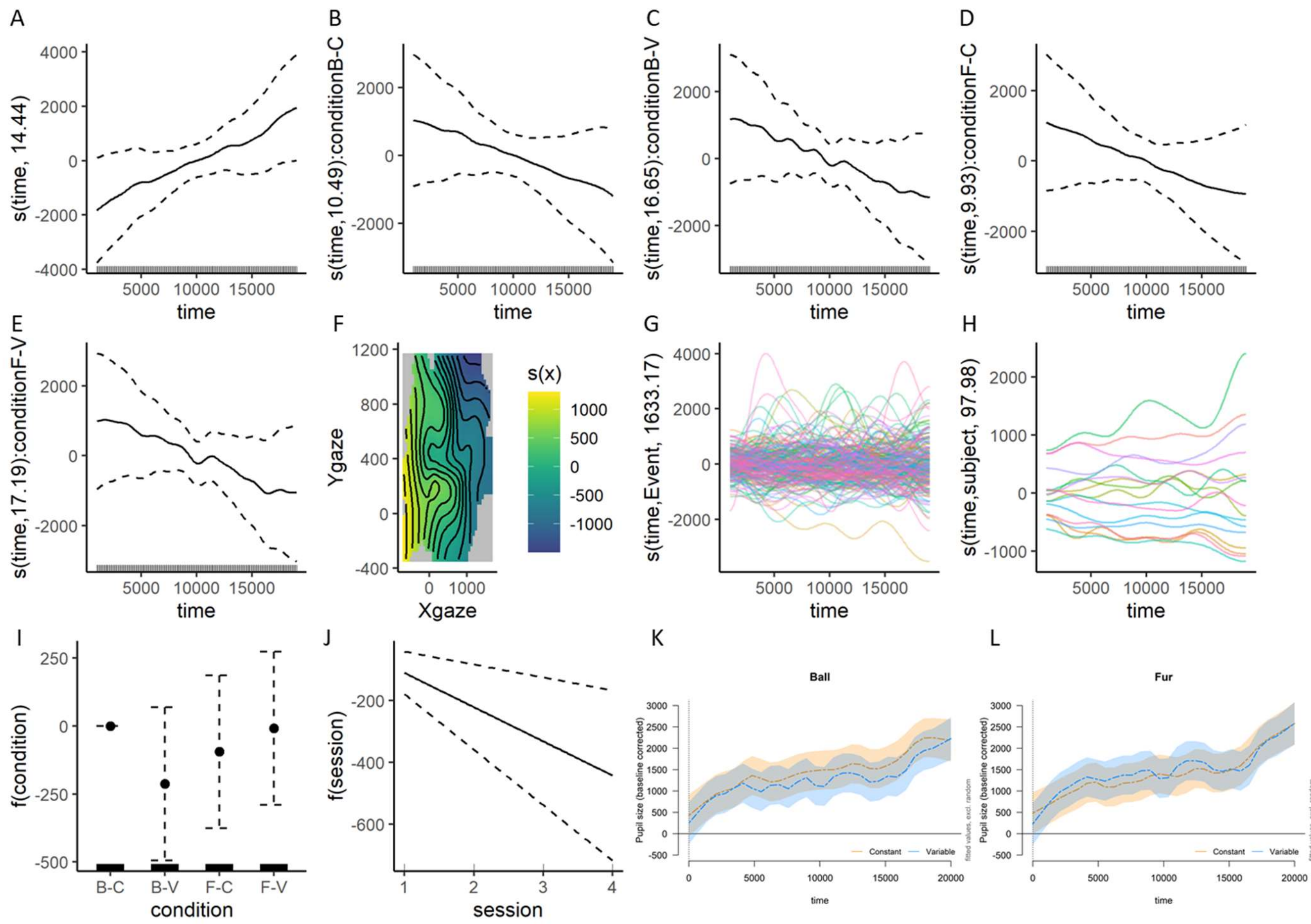


*Figure S8* Plots of the partial and summed effects of GAMM 05 (Experiment 2); related to Figure 4. All parametric and smooth components are plotted. A: smoothing term of time; B: Smoothing term of the Ball-constant condition over time; C: Smoothing term of the Ball-variable condition over time; D: Smoothing term of the Fur-constant condition over time; E: Smoothing term of the Fur-variable condition over time; F: Heatmap with overlaid contours of the 2D smooth of the X and Y gaze coordinates; G: random factor smooths for each trial and individual (N=204); H: random factor smooths for each individual (N=17); I: Parametric effect of condition. J. Parametric effect of session number (1-2). K: summed effects for the ball stimulus and both motion conditions (random effects set to 0). L: summed effects for the fur ball stimulus and both motion conditions (random effects set to 0). The partial effects plots have been created using the function `getViz` of the package mgcViz (Fasiolo et al., 2018). The summed effects plot has been created using the function `plot_smooth` of the package itsadug (van Rij et al., 2020).number. The plots have been created using the function `getViz` of the package mgcViz (Fasiolo et al., 2018).

*Table S11 Results of GAMM 05 (Experiment 2); related to Figure 4.*

| Parametric coefficients: | | | | |
|---|---|---|---|---|
| | Estimate | SE | t | p-value |
| (Intercept) | 1536.19 | 203.30 | 7.56 | <0.001 |
| ConditionBall-Variable | -174.84 | 142.43 | -1.23 | 0.220 |
| ConditionFur-Constant | -47.19 | 142.03 | -0.33 | 0.740 |
| ConditionFur-Variable | 84.96 | 142.53 | 0.60 | 0.551 |
| Session | -113.70 | 40.70 | -2.79 | 0.005 |
| Smooth terms | | | | |
| | edf | Ref.df | F | p-value |
| s(Time) | 4.79 | 5.60 | 0.56 | 0.701 |
| s(Time):ConditionBall-Constant | 5.84 | 7.15 | 0.55 | 0.831 |
| s(Time):ConditionBall-Variable | 14.75 | 16.61 | 4.81 | <0.001 |
| s(Time):ConditionFur-Constant | 10.23 | 12.26 | 1.72 | 0.037 |
| s(Time):ConditionFur-Variable | 13.37 | 15.29 | 5.15 | <0.001 |
| s(Xgaze,Ygaze) | 27.12 | 28.63 | 114.33 | <0.001 |
| s(Time,Event) | 1655.33 | 1826.00 | 179.82 | <0.001 |
| s(Time,Subject) | 60.97 | 152.00 | 1.54 | <0.001 |

Notes: Reference category of condition: Ball-Constant

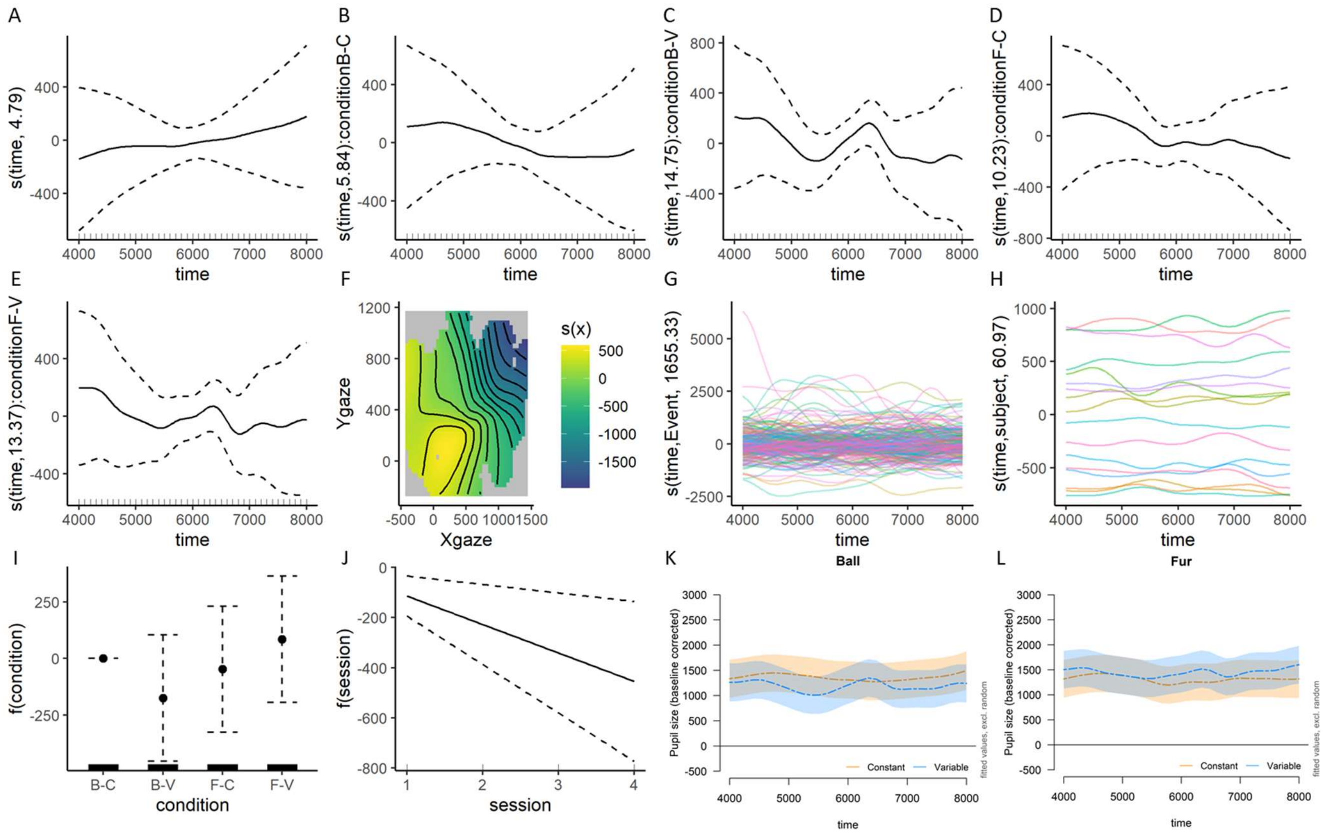


*Figure S9* Plots of the partial and summed effects of GAMM 05 (Experiment 2; first stop-start event per trial); related to Figure 4. All parametric and smooth components are plotted. A: smoothing term of time; B: Smoothing term of the Ball-constant condition over time; C: Smoothing term of the Ball-variable condition over time; D: Smoothing term of the Fur-constant condition over time; E: Smoothing term of the Fur-variable condition over time; F: Heatmap with overlaid contours of the 2D smooth of the X and Y gaze coordinates; G: random factor smooths for each trial and individual (N=204); H: random factor smooths for each individual (N=17); I: Parametric effect of condition. J. Parametric effect of session number (1-2). K: summed effects for the ball stimulus and both motion conditions (random effects set to 0). L: summed effects for the fur ball stimulus and both motion conditions (random effects set to 0).The partial effects plots have been created using the function `getViz` of the package mgcViz (Fasiolo et al., 2018). The summed effects plot has been created using the function `plot_smooth` of the package itsadug (van Rij et al., 2020).number. The plots have been created using the function `getViz` of the package mgcViz (Fasiolo et al., 2018).

**Supplementary references**

Fasiolo, M., Nedellec, R., Goude, Y., & Wood, S. N. (2018). Scalable visualisation methods for modern Generalized Additive Models. *Arxiv Preprint*. https://arxiv.org/abs/1809.10632

van Rij, J., Wieling, M., Baayen, R. H., & Rijn, H. van. (2020). *itsadug: Interpreting Time Series and Autocorrelated Data Using GAMMs* (R package version 2.4) [Computer software].
